# Effects of size and personality on social learning and human-directed behaviour in horses (*Equus caballus*)

**DOI:** 10.1007/s10071-019-01291-0

**Published:** 2019-07-16

**Authors:** Josefine Henriksson, Mathilde Sauveroche, Lina S. V. Roth

**Affiliations:** IFM Biology, AVIAN Behavioural Genomics and Physiology Group, Linköping University, 581 83 Linköping, Sweden

**Keywords:** Horse, Pony, Social learning, Contact-seeking behaviour, Human-directed behaviour, Interspecies communication

## Abstract

Due to our long history of living in close association with horses, these animals are suggested to have enhanced skills in understanding and communicating with humans. Today, horses have become important to humans for sport and leisure and their understanding of human behaviour and their human-directed behaviour are therefore of great importance. In this study, we investigated 22 horses in a human contact-seeking experiment where they were presented with an unsolvable problem and a detour experiment with a human demonstrator. The unsolvable problem consisted of pieces of carrot in a closed bucket and the detour resembled the shape of V. Additionally, personality traits of the participating horses were assessed. Interestingly, the full-sized horses (*N* = 11) showed more human-related behaviours when presented with an unsolvable problem compared to before the carrots were made unreachable (*p *= 0.033), while the ponies (*N* = 11) did not. However, neither the full-sized horses nor the ponies were significantly more successful in the detour after human demonstrations than in control trials. When comparing the two experiments, we found the task-oriented behaviour in the detour test to positively correlate with human proximity and eye contact-seeking behaviour towards humans during the unsolvable problem in the contact-seeking test. Interestingly, again this was only true for the full-sized horses (*p *< 0.05) and not for the ponies. From the horse personality questionnaire results, the traits excitability and anxiousness revealed strong negative correlations with human-directed behaviour in the contact-seeking experiment (*p *< 0.05). Hence, size (full-sized horse/pony) and personality influenced the human-related behaviours of the horses and we suggest a future focus on these aspects to deepen our understanding of human–horse communication.

**Electronic supplementary material:**

The online version of this article (10.1007/s10071-019-01291-0) contains supplementary material, which is available to authorized users.

## Introduction

Domestic animals, such as the horse (*Equus caballus*), have been living in close association with humans for thousands of years (Clutton-Brock 1981). Research has suggested that domestic animals have human-directed social skills that are not found in their wild ancestors. This has been particularly investigated in comparative studies between dogs and wolves (Gácsi et al. 2009; Miklósi et al. 2003; Virányi et al. 2008). For example, dogs have been shown to be sensitive to both the attention and emotions revealed by humans (D’Aniello et al. 2018; Müller et al. 2015; Virányi et al. 2004), to understand various referential gestures (Kaminski and Nitzschner 2013), and to learn from both conspecific and human demonstrators in social-learning tasks (Pongrácz et al. 2005; Scandurra et al. 2015). In addition, it is well established that dogs, contrarily to wolves, seek human contact when faced with a problem (Miklósi et al. 2003). This ability is evident at a young age and is age and breed dependent (Passalacqua et al. 2011; Sundman et al. 2017). Therefore, the domestication process appears to have facilitated interspecies communication abilities, not only in the context of understanding humans but also for the domesticated animal to communicate with humans. In this study we will investigate the interspecies communication abilities of the domestic horse.

Similar to dogs, horses show interspecies communication abilities with humans. For example, studies on horses suggest that they recognise the direction of human attention (Krueger et al. 2010; Proops and McComb 2009; Proops et al. 2013; Sankey et al. 2011) and human emotions (Lanata et al. 2018; Smith et al. 2016). However, their comprehension of human ostensive signals, such as pointing, is unclear and depends on the age of the horse and training method used (Dorey et al. 2014; Maros et al. 2008; Proops et al. 2010, 2013). Similarly, it was recently suggested that horses are not able to make use of information given by a human demonstrator in a detour task (Burla et al. 2018), even though they have been suggested to do so if the demonstrator is a conspecific (Krueger and Heinze 2008). However, a recent study (McVey et al. 2018) with refined methods found no difference in detour success rates between a control group and horses that had observed a demonstrating horse, similar to Dalla Costa et al. (2013) and Rørvang et al. (2015). Similarly, horses are suggested to be more successful at opening a feeding device after observing a human demonstrator (Schuetz et al. 2017); but again, there is a possibility that the horses learnt the task through trial and error. Hence, more research is needed to disentangle whether horses can acquire new information through social learning.

Horses have repeatedly shown the ability to seek human help when presented with an unsolvable task (Alterisio et al. 2018; Malavasi and Huber 2016; Ringhofer and Yamamoto 2016). Interestingly, horses communicate even more intensely if the human is not aware of the problem, suggesting that horses might alter their behaviour according to the humans previous attentional and knowledge state (Ringhofer and Yamamoto 2016). However, if the problem is solvable the horses that show more interest in a human experimenter are worse at solving the problem (Lesimple et al. 2012).

It is possible that human-directed behaviour and social abilities of horses could be influenced by different personality traits. For example, in dogs, individuals that score high on “sociability” gaze longer at a human experimenter during an extinction task (Jakovcevic et al. 2012). Indeed, it was recently suggested that personality traits might be one possible underlying reason for why horses vary in directional flexibility and their approach towards a spatial detour task (Baragli et al. 2017). In their detour task some horses were flexible when choosing direction and slower to reach the goal. And, if the detour was asymmetrical, they often selected the shorter way suggesting a reactive animal that slowly explores its surrounding and is therefore more accurate in its choices (Baragli et al. 2017). Other horses in the same study consistently detoured the barrier on the same side regardless of detour task which could suggest impulsiveness and proactive personality traits.

Research on human–horse interactions has, however, missed out on testing whether there is substantial variation between the different personalities of horses and their performances on tasks. In the current study, full-sized horses and ponies of different hierarchy ranks and sex participated in two different experiments investigating interspecies communication and social learning. Specifically, it was investigated whether they would seek contact with a passive human when presented with a problem and if they could make use of the information from an active human demonstrator in a detour task. Hence, in the first case the horse is the sender of the messages towards the human, in the other it acts as a receiver, reading human messages. Moreover, the link between these two abilities was assessed, i.e. if the horses that sought human contact also succeeded in a detour experiment after human demonstrations. Additionally, a horse personality questionnaire was completed for all participating horses to investigate whether there could be correlations between personality traits and human-directed behaviours.

## Method

The experiments were conducted at Smedstad riding centre in Linköping, Sweden. All 22 participating horses were riding school horses that attended riding classes and were fed as usual during this study. The age ranged from 7 to 25 years (13.5 years ± 0.9 SE) and included equal numbers of full-sized horses and ponies, mares and geldings and a balanced mix of what the staff at the riding centre reported was low, middle and high hierarchy ranked horses (Online Resource 1).

All horses included in the study were owned by the Smedstad riding centre. The management was informed about the study and gave their voluntary consent to participate with their full-sized horses and ponies. Hence, this study complies with the Swedish and European regulation for the use of animal subjects for research purposes. Since we only performed behavioural observations on these privately owned horses, no extra ethical permission was needed.

### Contact-seeking experiment

The aim of the contact-seeking experiment was to investigate whether the horses increased their human-related behaviours when they were presented with an unsolvable problem. The contact-seeking experiment was executed in one corner of an indoor arena (65 × 22 m) at Smedstad riding centre. The corner (15 × 16 m) was shielded off by jump stands and plastic wire. A food bucket was placed on a fence approximately 0.9 m above the ground and covered with a transparent Plexiglas lid with odour ports. All horses were familiar with the arena and were all tested separately with no visual contact with other horses.

**Fig. 1:**
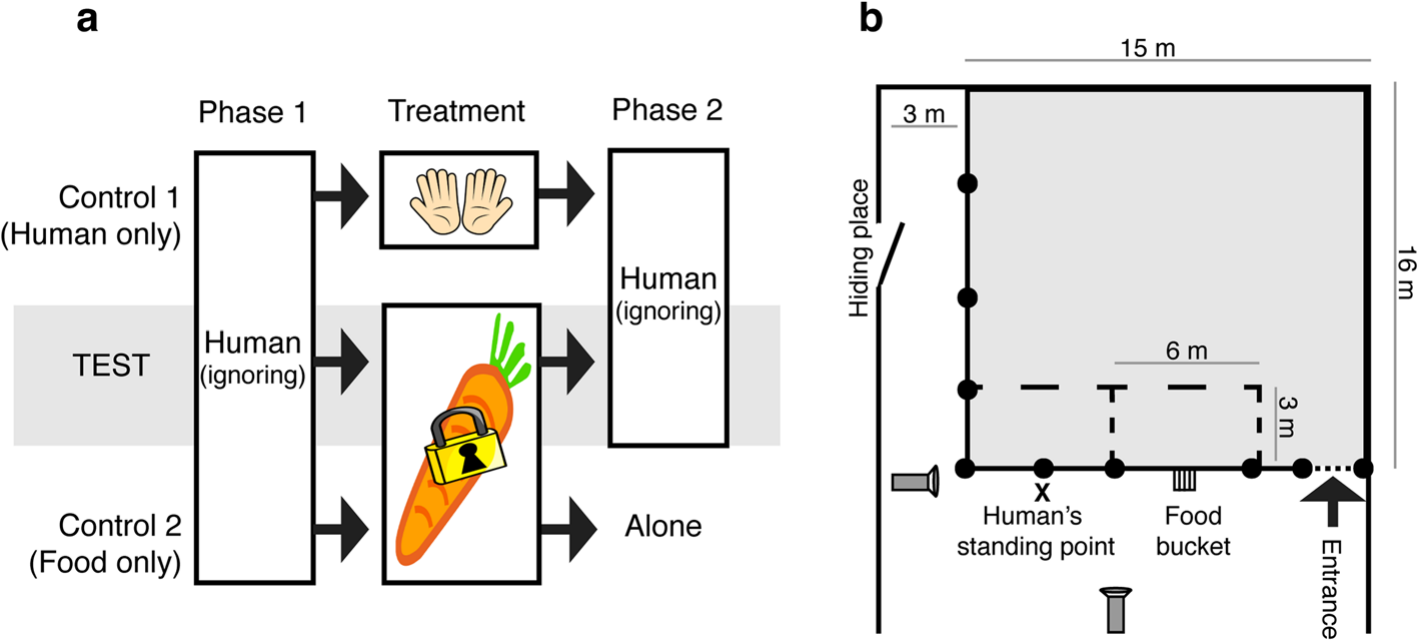
The contact-seeking experiment consisted of two controls and one test (**a**) and was conducted in one corner of an indoor arena (**b**). X indicates the human’s standing point, a striped square indicates the food bucket and two camera symbols indicate the position of the camcorders

The procedure was similar as described by Ringhofer and Yamamot (2016) and comprised two controls and one test. Each horse participated in all three trials on the same day in a predetermined order: control 1 (human only), test (human + food), and finally control 2 (food only) (Fig. 1a). The two controls were performed in order to evaluate if the presence of a human or food alone had an effect on the horse’s behaviour. When the horse entered the testing area for the very first time, it was given three pieces of carrot from the experimenter to test its food motivation. All horses ate the carrots and could continue on with the three trials. After the food motivation test, the experimenter left the test area for a hiding place to allow the horse to acclimatise to the new environment. The hiding place was behind a door 3 m away from the testing area and was used by both the experimenter and the helper during trials (Fig. 1b). The first control trial started after 5 min of acclimatisation time and there were 2-min breaks between the following two trials (before test and control 2).

Control 1 (human only)

- *Phase 1* The helper came out from the hiding place and entered a predetermined spot (“human’s standing point”) just outside the testing area. This spot was 6 m away from the food bucket (Fig. 1b). The helper was oriented 45° away from the food bucket and ignored the horse by checking her phone. The helper did not respond to any contact-seeking behaviours, like touching or looking, from the horse in order to prevent influencing the horse’s behaviour. After 1 min she returned to the hiding place.
- *Treatment* The experimenter came out from the hiding place, went to the food bucket and had physical interaction with the horse for a total of 10 s before returning to the hiding place.
- *Phase 2* The helper returned to the “human’s standing point” and again ignored the horse for 1 min before she returned to the hiding place. When this minute had passed, the experimenter came back and gave the horse three pieces of a carrot from her pocket.

Test (human + food)

- *Phase 1* Same as phase 1 in control 1 (human only).
- *Treatment* The experimenter came out from the hiding place, went to the food bucket and let the horse smell three pieces of carrot. The experimenter had physical interaction with the horse for a total of 10 s while standing next to the food bucket. Thereafter, she put the carrots in the bucket through the holes in the lid and left the arena.
- *Phase 2* Same as phase 2 in control 1, but this time the experimenter gave the horse the hidden pieces of carrot in the end of phase 2.

Control 2 (food only)

- *Phase 1* Same as in phase 1 in control 1 (human only).
- *Treatment* Same as in treatment in test (human + food).
- *Phase 2* The horse was left alone in the testing area. After 1 min both the helper and the experimenter entered the testing area and the helper gave the horse the hidden pieces of carrot.

#### Behavioural observations

Two HD camcorders (Canon Legria), recorded the behaviour of the horses during all trials (Fig. 1b). Behaviour items (Table 1) were recorded using a continuous sampling method and later phases 1 and 2 were compared within both control and test trials to see if there were any changes in the horses’ behaviours. In addition, behaviours in phase 1 of both controls and test trial were compared and similarly phase 2 for controls and test.

**Table 1:** Ethogram of behaviours recorded during contact-seeking experiment

Functional term	Descriptive term
Human proximity	The horse is standing still with at least one front hoof within 3 m from the helper
Food bucket proximity	The horse is standing still with at least one front hoof within 3 m from the food bucket
Elsewhere	The horse is not in proximity of either the human or the food bucket. The horse is instead in other parts of the testing area or walking in human or food bucket proximity areas
Looking at human	The horse’s head, eyes and at least one ear are oriented towards the helper
Looking at food bucket	The horse’s head, eyes and at least one ear are oriented towards the food bucket. Head/neck is extended towards the bucket
Looking at hiding place/door	The horse’s head, eyes and at least one ear are oriented towards the hiding place/door

### Detour demonstration experiment

The aim of the detour demonstration experiment was to assess whether the horse would be able to solve a simple detour task better after watching a human demonstrator. The contact-seeking experiment and the detour experiment were never conducted on the same day for any horse. All horses had finished the contact-seeking experiment before they were presented with the detour experiment in the same indoor arena. The experimenter in the contact-seeking experiment was the demonstrator in the detour experiment and the helper was the same as in the first experiment.

**Fig. 2:**
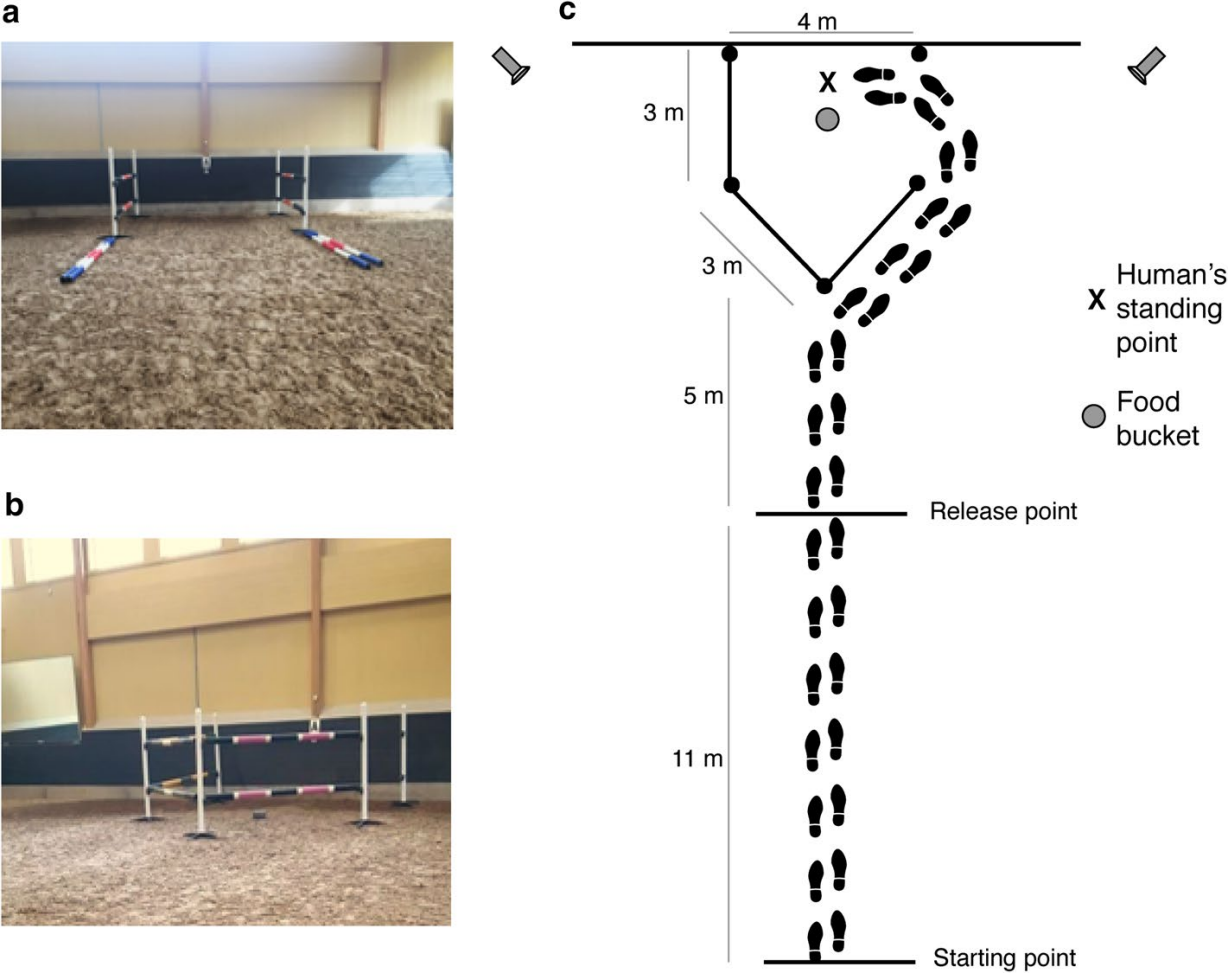
The detour experiment started with pretraining of the horse when the front part of the set-up was open (**a**). During controls and test the front part was closed and either the left or right side was opened (**b**). During test the human demonstrator repeatedly walked from the starting point to the human’s standing point (indicated with X) within the detour set-up which had either left or right side opened (**c**)

To begin with, each horse was led by the helper inside the indoor arena for 5 min to acclimatise to the environment. The horse was then led to the starting point, 16 m away from the detour set-up. The demonstrator gave the horse three pieces of carrot in a bucket, one at a time, as a test for food motivation. All horses ate the carrots and could continue on with the pre-training. The food bucket was moved by the demonstrator to inside the detour set-up which, during this pre-training, had the front part open (Fig. 2a). One piece of carrot was put into the bucket before the demonstrator called for the helper to lead the horse to the food bucket. This pre-training was repeated three times in order to make the horse familiar with the procedure and to associate the bucket with carrots.

After the pre-training, the detour set-up was rebuilt for the first control; the front part was closed and the opening was moved to either the left side (for half of the horses) or the right side (Fig. 2b). The horse was placed with its head in the opposite direction while the demonstrator entered the detour set-up through the middle part of the detour set-up to a predetermined “human’s standing point” (Fig. 2c). Then the horse was turned around to face the detour set-up. The demonstrator then stood still at the “human’s standing point”, between the bucket and the wall and dropped three pieces of carrot into the bucket. The demonstrator then called for the helper to lead the horse towards the detour set-up and let it loose at a predetermined “release point” 5 m away from the detour set-up. The horse then had 1 min to solve the task. In order to prevent any influence from the helper, this person looked down during the whole session.

For the demonstration test, the opposite side of the detour set-up was opened. This time, the demonstrator, the helper and the observing horse all started at the “starting point” (Fig. 2c). The demonstrator gave the horse one piece of carrot and then demonstrated the detour three times while talking to the horse. This was done in order to make the horse attentive with its head and at least one ear directed towards the demonstrator. One piece of carrot was placed in the food bucket within the detour set-up at the end of each demonstration. After the last demonstration the demonstrator stayed at the predetermined “human’s standing point”. Thereafter, the helper led the horse towards the set-up and released it at the predetermined “release point”. During all trials, the helper looked down and walked along a line in the dirt to prevent her from influencing the behaviour of the horse. The horse then had 1 min to find the opening. If it did not manage within this limited time, the horse was caught and returned to the starting point for a second trial with three new demonstrations. All horses had a maximum of three test trials for a maximum of nine demonstrations, but if a horse succeeded (at least one front leg through the opening in the fence), no more trials followed.

#### Behavioural observations

The main variable in the detour experiment was whether the horses made use of the information from the human demonstrations and succeeded in the detour test or not. Latency from release until the horse entered the detour set-up with its first front leg was also recorded. For example, if the horse succeeded 20 s into the second test trial the latency was 80 s, 60 s from the first trial plus 20 s from the second trial. The third and last trial was then never executed.

Two HD camcorder (Canon Legria) recorded the horse’s behaviour during all trials (Fig. 2c). Behaviour items (Table 2) were later recorded using a continuous sampling method.

**Table 2:** Behaviour items recorded during the detour experiment

Functional term	Descriptive term
Success	The horse entered the detour set-up with at least one front leg
Latency to success	The time from the first release until the horse enter the detour set-up with at least on front leg through the opening in the fence (max was 60 s per trial; total max after three test trials if unsuccessful was 180 s)
Task-oriented behaviour	Moving or standing still within 3 m from the detour set-up. At least one ear or the head directed towards the bucket/demonstrator

### Personality traits

The staff was asked to complete a horse personality questionnaire (HPQ) developed by Lloyd et al. (2007). The minimum requirement was that the person filling in the survey had known and handled the horse for at least 6 months. The HPQ consisted of 25 fully described adjectives (Online Resource 2) which were translated into Swedish and the staff was asked to score each horse from 1 to 7 for each adjective, where 1 meant no expression, 4 meant average and 7 meant total expression. The adjective scores for each horse were thereafter multiplied with the original PCA loadings (Lloyd et al. 2007) in order to achieve values on six different personality traits (Online Resource 1): Dominance (high negative loadings for reliability, subordinate, and equable and high positive loadings for irritable, aggressive, stubborn, effective, and eccentric), Anxiousness (high positive loadings for fearful, apprehensive, tense, insecure, and suspicious), Excitability (high negative loading for slow and high positive loading for active, intelligent, and excitable), Protection (high positive loadings for protective, motherly, and understanding), Sociability (high positive loadings for popular, playful, and sociable) and Inquisitiveness (high positive loadings for opportunistic and curious).

### Data analysis

Behavioural analysis from the video recordings was performed by the female experimenter. To assess the inter-rater reliability, the female acting as helper during the experiments recorded the behaviour from 10% of the horses in both experiments. The inter-rater reliability results showed high reliability for both experiments (contact-seeking exp.: rs = 0.97, *N* = 102; detour exp.: rs = 0.86, *N* = 42). Neither the experimenter nor the helper knew the hierarchy rank classifications or the personality assessment results of the horses when they performed the experiments or recorded the behaviours.

For data analysis, the horses were grouped according to their size (full-sized horse/pony). All statistical analyses were performed with SPSS (IBM version 24). Due to the non-parametrical nature of the behavioural data (Kolmogorov–Smirnov test), non-parametric tests were used. Differences between phases in the contact-seeking experiment were analysed with related-samples Wilcoxon signed rank tests and Friedman tests and the differences in success rate in the control and test phases of the detour experiment were tested with related-samples McNemar tests. Mann–Whitney U tests and Kruskal–Wallis tests were used to measure behavioural differences between two or more groups and Spearman rank correlations were used for correlations.

## Results

### Contact-seeking experiment

**Fig. 3:**
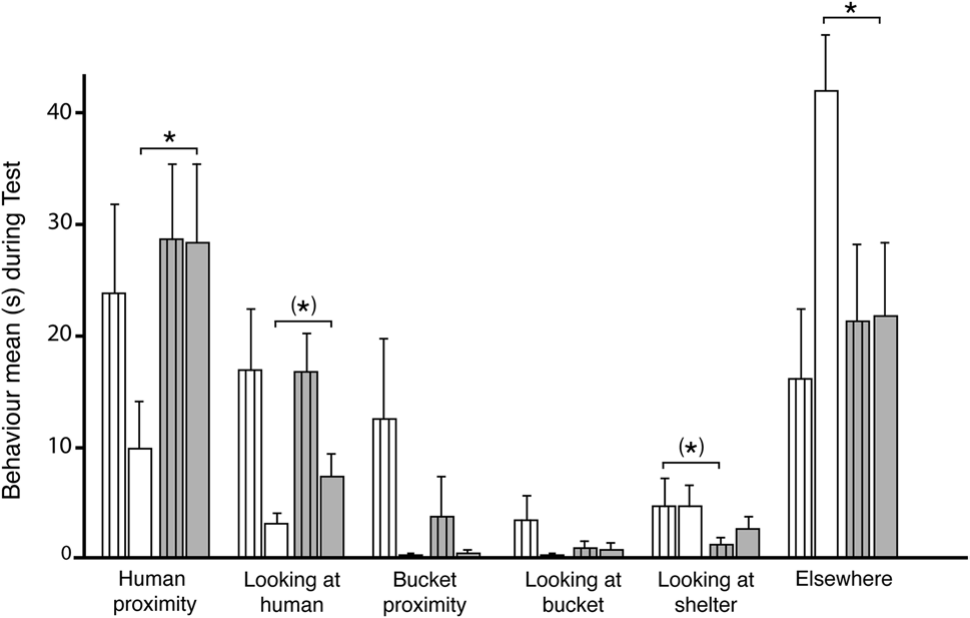
The behaviour items recorded during phase 1 (white bars) and phase 2 (grey bars) for both ponies (striped bars) and full-sized horses in the contact-seeking test (human + food). Asterisk in brackets indicates trends *p *< 0.1 and * indicates *p *< 0.05. Whiskers indicate 1 SE

Firstly, we compared phase 1 with phase 2 in the test (human + food; Fig. 3), i.e. the change in behaviour when the carrots were put in the food bucket. Interestingly, it was only the full-sized horses, and not the ponies, that revealed a significant increase of human proximity (*N* = 11, *Z* = − 2.13, *p *= 0.033) and also tended to look more towards human (*N* = 11, *Z* = − 1.89, *p *= 0.059) in phase 2 compared to phase 1. In line with this, full-sized horses spent significantly less time elsewhere in phase 2 compared to phase 1 (*N* = 11, *Z* = − 2.31, *p *= 0.021). No significant differences were found between test phases for ponies, but there was a tendency for the ponies to look less towards the shelter/door in phase 2 compared to phase 1 (*N* = 11, *Z* = − 1.76, *p *= 0.078).

**Fig. 4:**
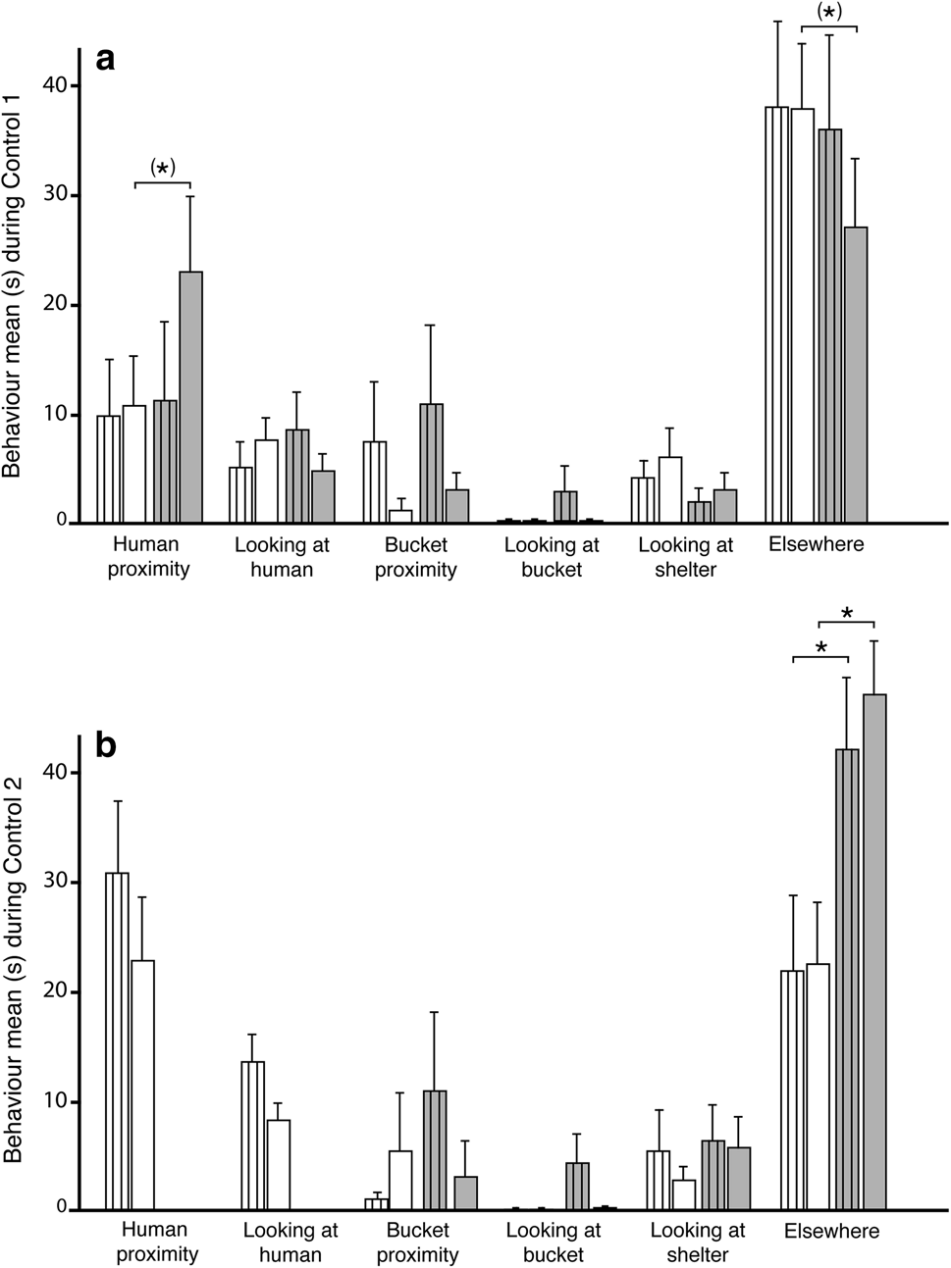
The behaviour items recorded during phase 1 (white bars) and phase 2 (grey bars) for both ponies (striped bars) and full-sized horses in **a** control 1 (human only) and **b** control 2 (food only) of the contact-seeking experiment. Asterisk in brackets indicates trends *p *< 0.1 and * indicates *p *< 0.05. Whiskers indicate 1 SE

During control 1 (human only; Fig. 4a), the full-sized horses, but not the ponies, showed a tendency to spend more time in human proximity in phase 2 after human interaction than in phase 1 (*N* = 11, *Z* = − 1.84, *p *= 0.066). Indeed, the full-sized horses also tended to decrease the time they spent elsewhere in phase 2 of control 1 (*N* = 11, *Z* = − 1.78, *p *= 0.075). During control 2 (food only; Fig. 4b) both full-sized horses and ponies spent significantly more time elsewhere in phase 2, i.e. when there was no human present, compared to phase 1 (*N* = 11, *Z* = − 2.22, *p *= 0.026 and *N* = 11, *Z* = − 2.37, *p *= 0.018, respectively).

There was a significant positive correlation between age and how much the horses looked at the food bucket in phase 2 during test (human + food; *N* = 22, rs = 0.49, *p *= 0.019). However, there was no significant correlation between age and total time looking at the food bucket (*N* = 22, rs = 0.22, *p *= 0.34) or if the horses were divided into full-sized horses and ponies.

Comparing human-related behaviours for the full-sized horses in phase 1 (before treatment; Online Resource 3) for both controls and test trial showed significant differences for human proximity behaviour (*N* = 11, *χ*^2^(2) = 7.34, *p *= 0.025) and time spent elsewhere (*N* = 11, *χ*^2^(2) = 9.22, *p *= 0.010). When phase 2 (after treatment; Online Resource 4) in control 1 and test trail were compared (there was no human present in phase 2 of control 2) there were no significant differences. Comparing both controls and test trial of phase 2 revealed a significant difference for time spent elsewhere (*N* = 11, *χ*^2^(2) = 9.15, *p *= 0.010).

Comparing human-directed behaviours for ponies in phase 1 (before treatment; Online Resource 3) for both controls and test trial did reveal a difference in their looking behaviour towards the helper (*N* = 11, *χ*^2^(2) = 7.43, *p *= 0.024) and there was also a tendency for the human proximity behaviour (*N* = 11, *χ*^2^(2) = 5.42, *p *= 0.067). In addition, there was a significant difference in the behaviour looking at food bucket (*N* = 11, *χ*^2^(2) = 7.05, *p *= 0.029) and a tendency for the horse to be elsewhere (*N* = 11, *χ*^2^(2) = 4.94, *p *= 0.085). When comparing phase 2 (after treatment; Online Resource 4) in control 1 and test trail (there was no human present in phase 2 of control 2) there was a significant difference for the behaviour looking at human (*N* = 11, *Z* = − 2.05, *p *= 0.041) and also a tendency for the human proximity behaviour (*N* = 11, *Z* = − 1.89, *p *= 0.059). Comparing both controls and test trial of phase 2 revealed a significant difference for the duration the horse spent elsewhere (*N* = 11, *χ*^2^(2) = 7.47, *p *= 0.024).

### Detour demonstration experiment

There was no significant difference in the success rate (i.e. entered the detour set-up with at least one front leg) between the full-sized horses and ponies after the human demonstrations compared to the initial control (*N* = 22, χ^2^(1) = 1.50, *p *= 0.22). While only one full-sized horse succeeded in the control, half of the full-sized horses succeeded in test; this did not, however, reach statistical significance (*N* = 11, χ^2^(1) = 2.25, *p *= 0.13; Table 3). Among the ponies, six succeeded in control 1 and five of these, plus one pony that failed in the control, mastered the task in test, after human demonstration (Table 3). Age did not differ between the successful and the unsuccessful horses in either the initial control or in the test (*N* = 22, *U* = 51.5, *p *= 0.56 and *N* = 22, *U* = 51, *p *= 0.95, respectively).

**Table 3:** Horses that succeeded or failed in the detour experiment

Individual	Sex	Size	Control 1	Test
Balou	Gelding	Pony	Success	Success
Lupin	Gelding	Pony	Success	Success
Tindra	Mare	Pony	Success	Success
Cilone	Mare	Pony	Success	Success
Larry	Gelding	Pony	Success	Success
Zunki	Mare	F-S horse	Success	Success
Pommac	Gelding	F-S horse	Fail	Success
Wilkette	Gelding	F-S horse	Fail	Success
Apple jack joe	Gelding	F-S horse	Fail	Success
Codoz	Gelding	F-S horse	Fail	Success
Colleen	Mare	Pony	Fail	Success
Mickel	Gelding	Pony	Success	Fail
Amorita	Mare	F-S horse	Fail	Fail
Cornelia	Mare	Pony	Fail	Fail
Felicia 3	Mare	Pony	Fail	Fail
Freja	Mare	Pony	Fail	Fail
Kipra	Mare	F-S horse	Fail	Fail
Livinga	Mare	F-S horse	Fail	Fail
Niko	Gelding	F-S horse	Fail	Fail
Qalypso	Gelding	F-S horse	Fail	Fail
Sachina	Mare	F-S horse	Fail	Fail
Totte	Gelding	Pony	Fail	Fail

*F*-*S  *full-sized

The horses that succeeded during the test after human demonstrations showed a higher proportion of task-oriented behaviour (*N* = 11, Mdn = 85%) than the unsuccessful ones (*N* = 11, Mdn = 9%; test: *N* = 22, *U* = 110.0, *p *= 0.001). There was no difference in task-oriented behaviour between ponies and full-sized horses during test (*N* = 22, *U* = 39.5, *p *= 0.17) and age did not correlate with task-oriented behaviour during the test (*N* = 22, rs = 0.14, *p *= 0.54).

### Comparing the behavioural experiments

Behaviour during phase 2 in the contact-seeking test (human + food), when the horse was presented with an unsolvable problem (unreachable carrots in food bucket), was compared to the detour test after human demonstrations. There were significant positive correlations found for human and interest-related behaviours in the two experiments. Dividing full-sized horses and ponies revealed that for the full-sized horses the task-oriented behaviour in the detour test correlated positively with human proximity and eye contact-seeking behaviour with the human (*N* = 11, rs = 0.83, *p *= 0.002; *N* = 11, rs = 0.69, *p *= 0.020) and with looking behaviour towards the bucket during phase 2 in the contact-seeking test (*N* = 11, rs = 0.61, *p *= 0.047). However, no significant correlations were found for the ponies (*N* = 11, *p *> 0.1).

Compared to the unsuccessful horses, the ones that were successful in the detour test did not spend more time in human proximity (*N* = 22, *U* = 60.5, *p *= 1.0) and did not seek more eye contact with human during the contact-seeking experiment (*N* = 22, *U* = 60.5, *p *= 1.0). Dividing into full-sized horses and ponies made no difference (*p *> 0.1).

### Personality traits

Investigating behaviours during phase 2 in the contact-seeking test (human + food) and their correlation to the personality of the horses (both full-sized horses and ponies) revealed significant negative correlations between the trait excitability and the behaviours human proximity, seeking eye contact with the human, and looking at the food bucket (Table 4). In addition, significant positive correlations were found between the trait excitability and being elsewhere and looking at the hiding place/door.

**Table 4:** Behaviour items from phase 2 in the contact-seeking test (human + food) and from the detour test (after human demonstrations) that correlated (*p *< 0.1) with personality traits (*N* = 22)

Behaviour	Excitability	Anxiousness	Protection	Dominance	Inquisitiveness	Sociability
*Contact-seeking test*						
Human proximity	**rs = − 0.49,** ***p *** **= 0.020**	rs = − 0.21, *p *= 0.35	rs = 0.37, *p *= 0.093	rs = − 0.41, *p *= 0.059	rs = − 0.40, *p *= 0.069	rs = 0.012, *p *= 0.96
Looking at human	**rs = − 0.63,** ***p *** **= 0.002**	**rs = − 0.43,** ***p *** **= 0.045**	rs = 0.22, *p *= 0.33	rs = − 0.19, *p *= 0.39	rs = − 0.24, *p *= 0.27	rs = − 0.23, *p *= 0.30
Looking at bucket	**rs = − 0.47,** ***p *** **= 0.026**	rs = − 0.099, *p *= 0.66	rs = 0.09, *p *= 0.69	rs = − 0.28, *p *= 0.21	rs = − 0.28, *p *= 0.21	rs = − 0.14, *p *= 0.55
Looking at door	**rs = 0.47,** ***p *** **= 0.029**	**rs = 0.64,** ***p *** **= 0.001**	rs = − 0.01, *p *= 0.97	rs = 0.14, *p *= 0.53	rs = 0.22, *p *= 0.32	rs = − 0.027, *p *= 0.91
Elsewhere	**rs = 0.53,** ***p *** **= 0.012**	rs = 0.37, *p *= 0.089	**rs = − 0.44,** ***p *** **= 0.042**	rs = 0.39, *p *= 0.070	rs = 0.39, *p *= 0.074	rs = − 0.098, *p *= 0.66
*Detour test*						
Task-oriented behaviour	**rs = − 0.68,** ***p *** **= 0.001**	**rs = − 0.45,** ***p *** **= 0.036**	rs = 0.24, *p *= 0.29	rs = − 0.052, *p *= 0.82	rs = − 0.27, *p *= 0.23	rs = − 0.013, *p *= 0.95

Significant results are shown in bold font

Similarly, the trait anxiousness correlated positively with looking towards the hiding place/door in phase 2 of the contact-seeking test (human + food) and correlated also negatively with the eye contact-seeking behaviour towards human (Table 4). On the contrary, the trait protection revealed a significant negative correlation with being elsewhere and showed a weak trend for spending more time in human proximity in phase 2 of the contact-seeking test (human + food).

Focusing on the phase 2 in contact-seeking test (human + food), there was a trend for a negative correlation between the dominance trait and human proximity (Table 4). In line with this, there was a trend for positive correlation between the trait dominance and the horse being elsewhere. Lastly, there was a tendency for negative correlation between the personality trait inquisitiveness and human proximity and also a trend for positive correlation for being elsewhere (Table 4).

Interestingly, there were significant negative correlations between the proportion of task-oriented behaviours during the detour test and the personality traits excitability and anxiousness (Table 4). However, there were no significant correlations between latency to succeed in the detour test (after human demonstrations) and the personality traits. Moreover, there were no significant differences in personality scores between those horses that succeeded and those that did not in either control 1, test, or control 2. However, there were weak trends for horses with high scores for inquisitiveness and dominance to succeed already in control 1 (*N* = 22, *U* = 27, *p *= 0.078 and *N* = 22, *U* = 28, *p *= 0.091, respectively). None of the behaviour items correlated significantly with the trait sociability (Table 4).

Comparing ponies and full-sized horses revealed that the full-sized horses scored higher than ponies on the sociability trait (*N* = 22, *U* = 97.0, *p *= 0.016). Testing the predetermined hierarchy rank (low, middle, high) scored by the riding school staff revealed significant results with the personality trait inquisitiveness (*N* = 22, rs = 0.43, *p *= 0.049). However, no other significant links were found between the predetermined rank and the personality traits from the survey (HPQ), or with any behaviour during phase 2 in the contact-seeking test, or with task-oriented behaviour and latency to success in the detour test (*p *> 0.1 in all cases).

## Discussion

The aim of this study was to investigate the human contact-seeking behaviour of horses and their social learning ability across species. In the present study, we found significant differences in human-directed behaviours between full-sized horses and ponies. The full-sized horses sought more human contact when presented with a problem, compared to before the carrots were put in the food bucket, which is in line with the previous studies (Alterisio et al. 2018; Malavasi and Huber 2016; Ringhofer and Yamamoto 2016). Interestingly, in our study the ponies did not differ in this comparison. In the detour test, half of the horses might have used the information from the human demonstrator. However, there was no significant difference between the initial control and the test, which is in accordance with a recent study (Burla et al. 2018).

To our knowledge, our study is the first to suggest differences between full-sized horses and ponies in their human contact-seeking behaviour. It was only the horses that significantly increased their human-related behaviours after they had been presented with an unsolvable problem. However, we did find increasing durations for human proximity and eye contact-seeking behaviour for both ponies and horses throughout the experiment. This was probably due to the carrots given to them after the controls and test trial. In contrast to the previous contact-seeking experiment (Alterisio et al. 2018; Malavasi and Huber 2016; Ringhofer and Yamamoto 2016), our study included a balanced number of both ponies and full-sized horses of different breeds, sex and hierarchy ranks. Ringhofer and Yamamoto (2016) included eight thoroughbreds which is a more homogenous population of full-sized horses only. In Malavasi and Hubber (2016) and Alterisio et al. (2018) different and mixed breeds were included but not further specified.

In the detour experiment, half of the ponies succeeded in both the initial control and in the test after human demonstration, while full-sized horses mainly succeeded after human demonstration. However, due to low number of horses this was not statistically confirmed. In Burla et al. (2018), neither breed nor size was specified for the 16 horses used for their detour experiment, but similarly to our detour experiment, their horses did not significantly improve after human demonstration. Likewise, they also used an unfamiliar demonstrator, which might have affected both our studies and will be discussed later.

When the two experiments, the contact-seeking experiment and detour experiment, in this study were compared, the horses that succeeded in the detour test after human demonstration did not show more human-related behaviour when presented with an unsolvable problem task in the contact-seeking experiment than those that did succeed in the detour test. Hence, if some individuals did make use of information from a human demonstrator this social ability was not linked to their human contact-seeking behaviour according to our results. This is similar to what is found in dogs where their ability to use human ostensive cues is not correlated to their human-directed behaviour in a problem-solving experiment (Sundman et al. 2017). It was therefore suggested that the two experiments measure different independently selected aspects of the dogs’ human-directed social behaviour. Still, we found positive correlations for the full-sized horses’ interest in the two experiments, i.e. human-related behaviours when presented with an unsolvable problem and task-related behaviours in the detour after human demonstration. But, again, there were no correlations for the ponies.

Personality traits are shown to be, at least to some extent, breed dependent (Lloyd et al. 2007). Full-sized horses are shown to be calmer than ponies during an open field test, while ponies are suggested to be less curious and attentive (Napolitano et al. 2008). These behavioural differences might be due to recent selective breeding of full-sized horses and ponies, which could be similar to behaviours in dogs that vary between recently selected breed lines (Sundman et al. 2016). However, there might also be differences in daily handling routines affecting the behaviour of full-sized horses and ponies, such as that the ponies have younger riders and handlers than the full-sized horses. Interestingly, it was only one personality trait, protection, that showed a weak tendency to correlate positively with a human-related behaviour (human proximity) in our contact-seeking experiment. Instead, almost all personality traits revealed negative correlations for human-related behaviours and this was especially pronounced for excitability and anxiousness. Moreover, there was no significant relationship between the personality traits and the horses’ success in the detour test. The lack of significant positive correlations with human-directed behaviours might simply be due to the shortage of questions on this subject within the HPQ. In many dog personality surveys there are questions dedicated to social behaviours towards humans, and, not surprisingly, there is a link between those behaviours and the dog’s trainability (Asp et al. 2015; Jones 2008). Dogs with high sociability score, determined during a behavioural test, gazed longer at a human experimenter (Jakovcevic et al. 2012). Similarly, the full-sized horses scored higher than ponies on sociability in our study and showed an increase in human-directed behaviour when presented with the unsolvable task. However, there was no correlation between the sociability trait and the horses’ human-related behaviours in the contact-seeking experiment. Future research should aim to include human-related questions in horse personality questionnaires to deepen our understanding of human–horse communication.

The task-oriented behaviours, which could be a measurement of interest and motivation for the detour experiment, revealed negative correlations with excitability and anxiousness. Similarly, these traits correlated positively with looking towards the hiding place/door in the contact-seeking experiment. Hence, horses with high scores for excitability and anxiousness were less motivated to participate in our experiments than other horses. Since the interest-related behaviours from the two experiments correlated this suggests that even though no personality trait correlated positively to any human-directed behaviour in this study, the motivation and interest to participate in the experiments were, at least to some extent, personality dependent.

In previous social learning experiments where horses have been demonstrators in detours for conspecifics, the dominance rank and familiarity have influenced the results but the results are contradictive. According to Krueger and Heinze (2008) horses can mimic the behaviour and follow a dominant and familiar conspecific. This is not supported by more recent studies (Dalla Costa et al. 2013; McVey et al. 2018; Rørvang et al. 2015). Even though Rørvang et al. (2015) showed a tendency for observing horses to be more successful in a detour experiment after demonstration, they were unable to repeat the results with a new group of horses and older demonstrators.

In previous problem-solving tasks, familiarity between demonstrator and observer did not improve the social learning in horses (Ahrendt et al. 2012), even though the authors acknowledged that the fear of aggressive encounters might have limited the observer to optimally learn the task in their study. In our experiments, the horses had only met the human experimenter and helper once. Although the horses in this study were active riding school horses that are handled by many different people, the fact that the humans in this study were relatively unfamiliar might have influenced the behaviour and the final result. This could also be true for the contact-seeking experiment. However, Alterisio et al. (2018) did not find any differences in the horses’ human-directed behaviours towards the male riding schools’ caretakers and a female stranger but the sex difference was not considered. Privately owned horses and their owners have yet to be tested, and this type of research could lead to important insights about how familiarity may influence a horse’s behaviour during these tasks. Future studies should investigate whether horses are more likely to make use of information given by their owners and show more human-directed behaviours towards them instead of a stranger. Another aspect to evaluate is whether there are more suitable experiments to investigate social learning, since horses might be, due to personality traits, more or less flexible in their approach to spatial tasks (Baragli et al. 2017).

In conclusion, in this study we showed that full-sized horses, but not ponies, showed more human-related behaviours when presented with an unsolvable problem, and we found several correlations between these behaviours and personality traits. In the detour, neither full-sized horses nor ponies seemed to make use of the information from a human demonstrator. However, there were positive correlations between human-directed behaviours in the contact-seeking experiment and task-related behaviours in the detour experiment for the full-sized horses, but not for ponies. Hence, we show that size and personality have an impact on the human-related behaviour in both experiments.

## Electronic supplementary material

Below is the link to the electronic supplementary material.

Supplementary material 1 (XLSX 22 kb)

Supplementary material 2 (DOCX 15 kb)

Supplementary material 3: **Fig Online Resource 3.** The behaviour items recorded for full-sized horses (**a**) and ponies (**b**; striped bars) during phase 1 (before treatment) in control 1 (C1), test (*T*) and control 2 (C2) in the contact-seeking experiment. Asterisk in brackets indicates trends *p *< 0.1 and * indicates *p *< 0.05. Whiskers indicate 1 SE (TIFF 875 kb)

Supplementary material 4: **Fig Online Resource 4.** The behaviour items recorded for full-sized horses (**a**) and ponies (**b**; striped bars) during phase 2 (after treatment) in control 1 (C1), test (*T*) and control 2 (C2) in the contact-seeking experiment. Asterisk indicates *p *< 0.05. *Z* indicates statistical difference between two groups (due to the absence of human in C2), where (*Z*) indicates trend *p *< 0.1 and *Z* indicates *p *< 0.05. Whiskers indicate 1 SE (TIFF 859 kb)

## Data Availability

Data analysed during this study are included in this published article and its supplementary information files.
