# Supplementary material for: Effects of size and personality on social learning and human-directed behaviour in horses (Equus caballus)
Source: Anim Cogn. 2019 Jul 16;22(6):1001–11. doi: 10.1007/s10071-019-01291-0 (PMC6834737; doi:10.1007/s10071-019-01291-0)
Supplement: Supplementary file 2 — Supplementary material 2 (DOCX 15 kb) [file 10071_2019_1291_MOESM2_ESM.docx]

**Effects of size, sex and personality on social learning and human directed behaviour in horses (Equus caballus)**

*Animal Cognition*

Josefine Henriksson, Mathilde Sauveroche, L.S.V Roth*
IFM Biology, AVIAN Behavioural Genomics and Physiology group, Linköping University, 581 83 Linköping, Sweden.*Corresponding author: lina.roth@liu.se, ORCID: 0000-0002-3297-1130

**Supplementary 2. Adjectives and definitions used in the horse personality questionnaire described by Lloyd et al. (2007)**

| **Adjective** | **Description** |
| --- | --- |
| **Reliable** | Can be trusted to do things or behaves well might also be considered a safe horse to be with. |
| **Subordinate** | Gives in readily to others, submits easily and does not put up a fight to defend self. |
| **Equable** | Reacts to others in an even, calm way; not easily disturbed. |
| **Eccentric** | Shows stereotypes, unusual mannerisms and exaggerated behaviour. |
| **Effective** | Gets own way, can control others, fairly dominant individual. |
| **Stubborn** | Does not give in easily, not very cooperative. |
| **Aggressive** | Causes harm or potential harm to other individuals, both horse and human. |
| **Irritable** | Reacts negatively with little provocation. |
| **Suspicious** | Does not trust others readily (human and horse), trusts few individuals. |
| **Insecure** | Hesitates to act alone; seeks reassurance from others. |
| **Tense** | Shows restraint in posture and movement; carries the body stiffly, which suggests a shrinking tendency, as if to pull back and be less conspicuous. |
| **Apprehensive** | Seems to be anxious about everything, fears or avoids any kind of risk. |
| **Fearful** | Retreats readily from others or from outside disturbances. |
| **Active** | Moves around a lot, does not like being still for long. |
| **Slow** | Moves and rests in a relaxed manner, moves slowly and deliberately, not easily hurried. |
| **Excitable** | Over reacts to any change, easily excited, highly strung. |
| **Intelligent** | Learns new things easily/fast benefits from mental stimulation. |
| **Understanding** | Responds in a discriminating and appropriate manner to the behaviour of others. |
| **Motherly** | Provides warm receptive secure base for others, is tender and caring. |
| **Protective** | Prevents harm or possible harm to others. |
| **Sociable** | Seeks companionship of others. |
| **Playful** | Initiates play and joins in when play is solicited. |
| **Popular** | Sought out as a companion by others. |
| **Curious** | Readily explores new situations. |
| **Opportunistic** | Seizes a chance as soon as it arises. |
