# Supplementary figures and images for: Effects of size and personality on social learning and human-directed behaviour in horses (Equus caballus)
Source: Anim Cogn. 2019 Jul 16;22(6):1001–11. doi: 10.1007/s10071-019-01291-0 (PMC6834737; doi:10.1007/s10071-019-01291-0)

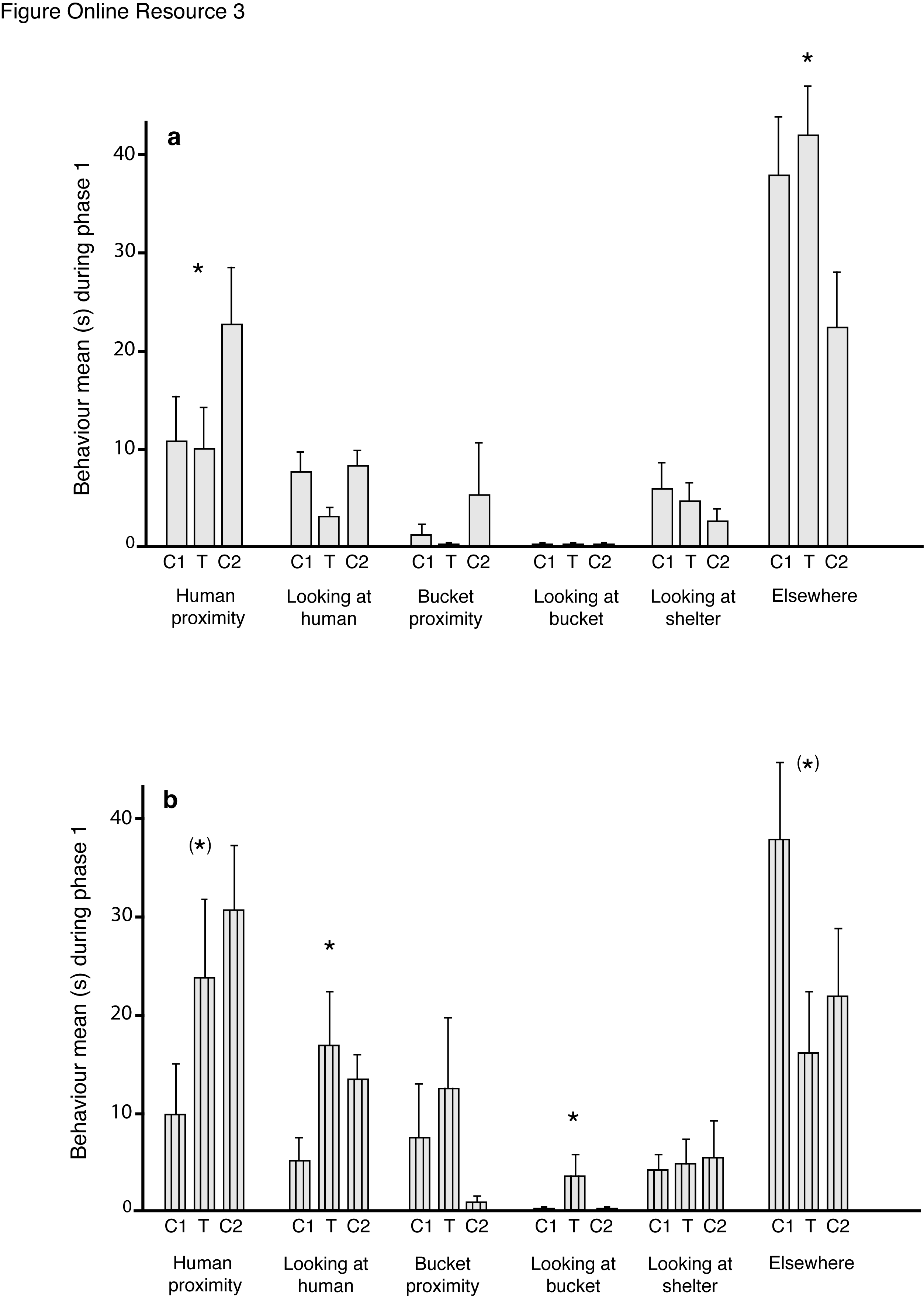

Supplement: Supplementary file 3 — Supplementary material 3: Fig Online Resource 3. The behaviour items recorded for full-sized horses (a) and ponies (b; striped bars) during phase 1 (before treatment) in control 1 (C1), test (T) and control 2 (C2) in the contact-seeking experiment. Asterisk in brackets indicates trends p < 0.1 and * indicates p < 0.05. Whiskers indicate 1 SE (TIFF 875 kb) [file 10071_2019_1291_MOESM3_ESM.tif]

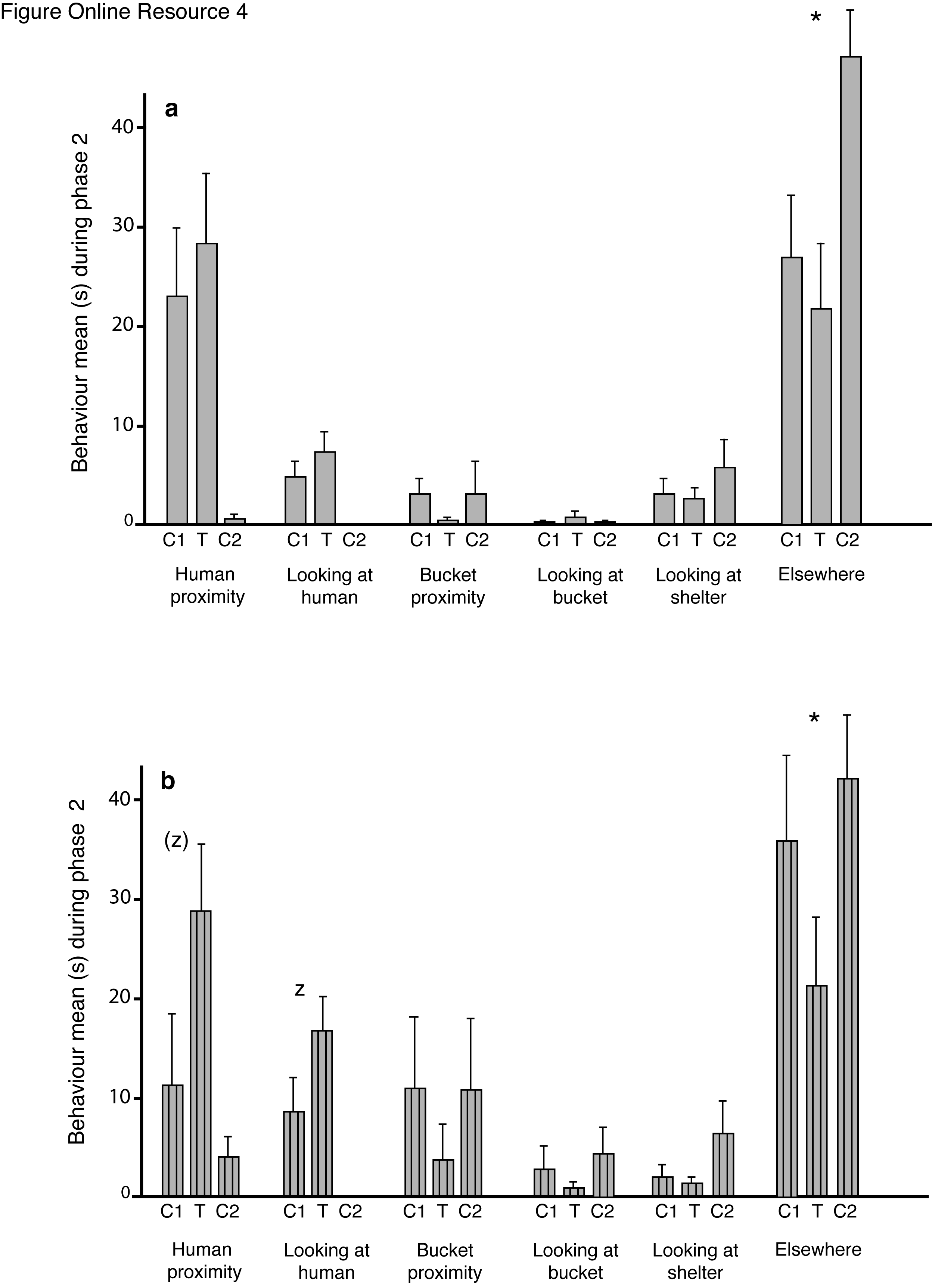

Supplement: Supplementary file 4 — Supplementary material 4: Fig Online Resource 4. The behaviour items recorded for full-sized horses (a) and ponies (b; striped bars) during phase 2 (after treatment) in control 1 (C1), test (T) and control 2 (C2) in the contact-seeking experiment. Asterisk indicates p < 0.05. Z indicates statistical difference between two groups (due to the absence of human in C2), where (Z) indicates trend p < 0.1 and Z indicates p < 0.05. Whiskers indicate 1 SE (TIFF 859 kb) [file 10071_2019_1291_MOESM4_ESM.tif]
